# Patients with DVT and primary antiphospholipid syndrome have worse obstetric outcomes than pregnant women with DVT and negative antiphospholipid antibodies: a retrospective cohort study

**DOI:** 10.1590/1516-3180.2024.0310.R1.24032025

**Published:** 2025-08-29

**Authors:** Priscila Guyt Rebelo, Marcela Ignacchiti Lacerda Ávila, Nilson Ramires de Jesús, Flávio Victor Signorelli, Evandro Mendes Klumb, Guilherme Ramires de Jesús

**Affiliations:** I Physician, Departamento de Obstetrícia, Hospital Universitário Pedro Ernesto, Universidade do Estado do Rio de Janeiro (UERJ), Rio de Janeiro (RJ), Brazil.; II Physician, Departamento de Obstetrícia, Hospital Universitário Pedro Ernesto, Universidade do Estado do Rio de Janeiro (UERJ), Rio de Janeiro (RJ), Brazil.; III Physician, Departamento de Obstetrícia, Hospital Universitário Pedro Ernesto, Universidade do Estado do Rio de Janeiro (UERJ), Rio de Janeiro (RJ), Brazil.; IV Physician, Departamento de Reumatologia, Hospital Universitário Pedro Ernesto, Universidade do Estado do Rio de Janeiro (UERJ), Rio de Janeiro (RJ), Brazil.; V Physician, Departamento de Reumatologia, Hospital Universitário Pedro Ernesto, Universidade do Estado do Rio de Janeiro (UERJ), Rio de Janeiro (RJ), Brazil.; VI Physician, Departamento de Obstetrícia, Hospital Universitário Pedro Ernesto, Universidade do Estado do Rio de Janeiro (UERJ), Rio de Janeiro (RJ), Brazil.

**Keywords:** Venous thrombosis, Antiphospholipid syndrome, Pregnancy complications, Thrombophilia, Antibodies, antiphospholipid, Thrombosis, Lupus coagulation inhibitor, Antibodies, anticardiolipin, Pre-eclampsia, Anticoagulants

## Abstract

**BACKGROUND:**

Pregnant women are at an increased risk of thromboembolism compared with non-pregnant women. Venous thrombosis is a manifestation of antiphospholipid syndrome (APS), an autoimmune thrombophilia associated with pregnancy morbidity.

**OBJECTIVES:**

This study was designed to compare gestational outcomes of pregnant patients with deep venous thrombosis (DVT) and primary APS with outcomes of patients with DVT and negative results for antiphospholipid antibodies (aPLs).

**DESIGN AND SETTING:**

This was a retrospective cohort study with data collected from patients with DVT who received prenatal care for autoimmunity and thrombophilia at Hospital Universitário Pedro Ernesto, Rio de Janeiro.

**METHODS:**

All patients with DVT were tested for aPLs. Those with positive results were tested again after 12 weeks and classified as having primary APS. Patients with systemic lupus erythematosus, superficial venous thrombosis without DVT, twin pregnancies, or fetuses with congenital malformations were excluded.

**RESULTS:**

This study included 171 patients (39 with APS, 132 with DVT and negative aPL results). Patients with primary APS and DVT had higher frequencies of miscarriages (P = 0.004) and stillbirths during previous pregnancies (P < 0.001). When obstetric outcomes were analyzed prospectively, APS patients had a lower birth weight (P = 0.001) and higher rates of oligohydramnios (P = 0.04), intrauterine growth restriction (P = 0.01), preeclampsia (P = 0.04), stillbirths (P = 0.02), and small-for-gestational-age newborns (P < 0.001) than patients with DVT and negative aPL results. The latter group had gestational outcomes similar to those of the general population.

**CONCLUSIONS:**

Patients with primary APS have adverse obstetric outcomes despite appropriate treatment, whereas those with DVT and negative for aPLs have favorable results.

## INTRODUCTION

 Pregnant women have four times the risk of thromboembolism compared with non-pregnant women.^1^ Among other risk factors, pregnancy is a hypercoagulable condition preparatory to childbirth, and compression of the inferior vena cava by the gravid uterus contributes to venous stasis, thus favoring thrombotic phenomena.^1^

 Venous thrombosis is a manifestation of antiphospholipid syndrome (APS), an autoimmune disease associated with vascular manifestations and circulating antiphospholipid antibodies (aPLs). It is estimated that the overall aPL frequency in patients with deep venous thrombosis (DVT) is 9.5% and, as approximately 70% of individuals with APS are female, this disease is found in women of reproductive age.^2^

 Pregnancy morbidity is also a well-recognized presentation of APS and is part of the classification criteria.^3^ Women with persistent aPLs have a higher incidence of recurrent abortions, fetal losses, preeclampsia (PE), and placental insufficiency than the general population.^3^ These events were also previously associated with hereditary thrombophilias; however, recent literature questions whether this relationship really exists.^4^

## OBJECTIVES

 This study aimed to compare the gestational outcomes of pregnant patients with a history of or current DVT and primary APS with outcomes of patients with DVT and negative results for aPLs. 

## METHODS

 This was a cohort study with retrospective data collection of patients with DVT that occurred before or during pregnancy, who received prenatal care for autoimmunity and thrombophilia at Hospital Universitário Pedro Ernesto, Rio de Janeiro, since 2005. All patients had DVT as an entry criterion and were tested for aPLs at least once, while patients with positive aPL results were retested after 12 weeks to confirm primary APS classification.^3^ Patients were not routinely tested for inherited thrombophilia as it would not change the recommended treatment according to current protocols. Patients with systemic lupus erythematosus, superficial venous thrombosis without DVT, twin pregnancies, or fetuses with congenital malformations were excluded from the analysis. 

 Clinical manifestations, demographic and obstetric characteristics, gestational results, and prescribed treatments were organized in tables and subsequently submitted for descriptive statistical analysis. Fisher’s exact test was used to compare categorical variables, and the Mann–Whitney U test was used for continuous variables that did not show a normal distribution. This study was approved by the local institutional Ethics Committee of Hospital Universitário Pedro Ernesto, Rio de Janeiro (approval number: 02190912.6.1001.5259). 

## RESULTS

 One hundred eighty-five pregnancies in 171 patients with a history of DVT were evaluated. Of these, 39 fulfilled the APS criteria (Group 1) and 132 were aPL-negative (Group 2). Patients with APS had a higher mean age (32.4 ± 5.2 versus 29.6 ± 6.4, P = 0.006) and more previous stillbirths (23% versus 2%, P < 0.001) and miscarriages (43.5% versus 20.45%, P = 0.004) (Table 1). Another issue identified was the diagnosis of thrombosis (7.6% versus 21.2%, P = 0.02) during this pregnancy. Among the comorbidities, the more frequent were systemic arterial hypertension (7.6% versus 86%, P = 0.3), type 2 diabetes (5.1% versus 3.0%, P = 0.2), and gestational diabetes (5.1% vs. 7.5%, P = 0.3). Among patients with DVT, 9.8% had already received treatment (full-dose anticoagulation) at the beginning of pregnancy. 

**Table 1:** Characteristics of patients with DVT who received prenatal care for autoimmunity and thrombophilia at Hospital Universitário Pedro Ernesto, Rio de Janeiro

**Variable**		**PAPS (n = 39)**	**aPL negative (n = 132)**	**P value**
Age at birth (years)				
	*Mean ± SD*	32.4 ± 5.2	29.6 ± 6.4	**0.006**
	*Min–Max*	21–42	15–44	
Abortion history				
	*N (%)*	17 (43.5%)	27 (20.45%)	**0.004**
Background of stillbirth				
	*N (%)*	9 (23%)	2 (1.5%)	**0.00002**
Trombosis in current pregnancy				
	*N (%)*	3 (7.6%)	28 (21.2%)	0.02
Comorbidities				
	*Arterial Hypertension*	3 (7.6%)	8 (6%)	0.3
	*Type 2 Diabetes*	2 (5.1%)	4 (3%)	0.2
	*Gestational Diabetes*	2 (5.1%)	10 (7.5%)	0.3
	*Others*	4 (10.2%)	5 (3.7%)	0.07
DVT				
	*Lower limbs*	32 (82%)	119 (90.1%)	0.1
	*Chronic PE*	1 (2.5%)	3 (2.2%)	0.6
	*Other locations*	6 (15.3%)	13 (9.8%)	0.1
Immunological profile		N		
	*LAC*	22 (56%)	N/A	
	*ACL*			
	*IgM*	5 (12.8%)	N/A	
	*IgG*	11 (28.2%)	N/A	
	*AntiB2*			
	*IgM*	3 (7.6%)	N/A	
	*IgG*	6 (15.3%)	N/A	
Treatment				
	*Aspirin use (with or without anticoagulant)*	32 (82%)	76 (57.5%)	**0.003**
	*No anticoagulant*	0	8 (6%)	0.06
	*Prophylatic anticoagulant only*	2 (5.1%)	31 (23.4%)	**0.003**
	*Prophylatic anticoagulant plus aspirin*	2 (5.1%)	50 (37.8%)	**< 0.0001**
	*Full-dose anticoagulant only*	5 (12.8%)	22 (16.6%)	0.29
	*Full-dose anticoagulant plus Aspirin*	30 (76.9%)	21 (15.9%)	**< 0.0001**

PAPS: primary antiphospholipid syndrome; aPL: antiphospholipid antibody; DVT: deep venous thrombosis; N/A: not applicable.

 The most frequent autoantibody in Group 1 was lupus anticoagulant (56%), with three triple-positive patients. In both groups, the most common site of DVT was the lower limbs (82% and 90.1%, respectively). All patients in Group 1 used anticoagulants during pregnancy, with a predominance of full anticoagulation (89.7%, P < 0.001), and 82% used low dose aspirin (LDA), while 61.3% of Group 2 used prophylactic anticoagulants (P < 0.001), and 57.5% used LDA (P = 0.003). 

 Among adverse obstetric outcomes, patients with APS had worse gestational results (Table 2), such as higher rates of oligohydramnios (10.2% versus 2.2%, P = 0.04), PE (15.3% versus 5.3%, P = 0.04), and intrauterine growth restriction (IUGR) (17.9% versus 5.3%, P = 0.04), and lower birthweight (2,877 g ± 548 versus 3,225 g ± 565, P = 0.001) when compared with aPL-negative patients. Two cases of intrauterine fetal death occurred in the APS group. There were no differences between the two groups in terms of the route of delivery, 5 min Apgar score, admission to the neonatal intensive care unit, and premature delivery. 

**Table 2:** Details of delivery, newborns, and adverse obstetric outcomes of patients with DVT

**Variable**		**PAPS (n = 39)**		**aPL negative (n = 132)**		**P value**
Delivery		n	%	n	%	P
	*Vaginal*	13	33.3	59	44.6	0,1
	*C-section*	26	66.6	73	55.3	0.1
Gestational age (weeks)		n = 37*				
	*Mean ± SD*	37.6 ± 1.5		38.1 ± 1.7		0.08
Newborn weight		n = 37*				
	*Mean ± SD*	2877 ± 548		3225 ± 565		0.001
Small for gestational age		n = 37*				
		7	18.9%	6	4.5%	0.008
5 min Apgar score		n = 37*				
		8.9	± 1.1	8.8	± 0.7	0.6
Admission to NICU		n = 37*				
		5	13.5%	16	12.1%	0.5
Oligohydramnios		4	10.2	3	2.2	0.04
Intrauterine growth restriction		7	17.9	7	5.3	0.01
Premature rupture of membranes		3	7.6	17	12.8	0.2
Preeclampsia		6	15.3	7	5.3	0.04
Premature delivery		7	17.9	15	11.3	0.2
Stillbirth and neonatal death		2	5.1	0	-	0.02
Acute fetal distress		7	17.9	13	9.8	0.1

DVT: deep venous thrombosis; PAPS: primary antiphospholipid syndrome; aPL: antiphospholipid antibody; NICU: neonatal intensive care unit; * Two stillbirths were excluded.

## DISCUSSION

 Obstetric complications such as fetal death, preterm birth, PE, and recurrent early miscarriage (REM) are characteristic manifestations of APS. These complications can occur in patients with known APS with previous arterial or venous events in any tissue or organ, or they can be the first and only manifestations. Pregnancy in patients with APS is considered high risk and requires careful prenatal clinical follow-up, eliminating or minimizing concomitant thrombotic risk factors.^5^

 The current recommended treatment for APS patients and vascular thrombosis is LDA plus full-dose heparin throughout pregnancy.^5^ Although treatment can significantly improve live birth rates, the frequency of adverse obstetric outcomes and even pregnancy loss remains high.^1^ In this scenario, there is no study to support the use of any different drug, and proposed treatments rely on expert opinion. New pathogenic mechanisms of aPL-mediated pregnancy loss have been investigated in recent years, and this may be helpful in finding specific targets for future treatment proposals for refractory cases.^6^ The main question is whether the treatment can prevent the development of PE and intrauterine growth restriction in aPL-positive patients. 

 The actual frequency of PE in treated APS patients is still unknown, but there are reports ranging from 14% to as high as 50%, with half of the cases being severe. This rate is considerably higher than that seen in the general population, estimated up to 7%.^6^ In this study, 15% of APS patients developed PE, despite the fact that more than 80% used LDA. On the other hand, patients with DVT and negative aPL results developed PE in 5.3% of pregnancies, a frequency that is similar to that reported in Brazil.^7^

 This high frequency, despite treatment, may represent a different pathway than that of non-aPL-related hypertensive disorders of pregnancy. For example, LDA, recommended as standard-ofcare for APS during pregnancy, reduced the occurrence of PE in 53% of non-APS high-risk patients when started before 16 weeks of pregnancy, with a reduction of almost 80% in severe cases.^8^ While studies in APS patients are still lacking, we do not seem to be closer to those numbers with current treatment. 

 IUGR is another placenta-mediated event associated with APS. An accurate diagnosis for this condition is still lacking in the obstetric field, but the usual definition is based on estimated fetal weight below the 10th percentile.^9^ The described rate of IUGR in APS patients varies from 15% to 21% in the literature, which is similar to our findings, but there are reports of almost 40% of small for gestational age neonates in patients with thrombotic APS.^6^ In addition to PE, IUGR is commonly found in pregnant women with APS before and after treatment. Both diseases represent different clinical manifestations of abnormal placental function and can occur in the same patient. However, isolated fetal impairment is possible and may develop with absent or minimal maternal signs, such as a small uterine height. Therefore, it is recommended that monthly ultrasound evaluation and Doppler velocimetry studies be performed routinely for APS patients, starting after 26 weeks of pregnancy.^5^

 Again, the treatment for this presentation of obstetric APS is disappointing. In this study, pregnant patients with APS had more IUGR and newborns with lower birthweight than aPL-negative patients. Our results are similar to those of previous studies that evaluated obstetric outcomes in APS patients and demonstrated the difficulty in reducing pregnancy morbidity in this group.^10^

 It is also important to note that preterm birth is the most frequent neonatal complication of APS during pregnancy, affecting between 20% and 25% of patients,^6^ and is mainly caused by medical intervention because of PE and/or IUGR. Preterm delivery is required in approximately one-third of APS patients with PE,^6^ while spontaneous premature birth is less frequent. In this study, the patients with APS had a higher number of preterm births; however, the difference was not statistically significant. As the frequencies of PE and IUGR remain high in women with APS, it is difficult to determine an exact estimate of spontaneous preterm births in these patients. Specific treatment to prevent premature birth is not recommended as it is a common consequence of other obstetric complications. 

 In addition to APS, hereditary thrombophilia is another common cause of DVT. This type of thrombophilia is defined as the tendency for thrombosis due to hereditary alterations (protein C, S, and antithrombin deficiencies; factor V Leiden; and prothrombin gene mutations). There are inconsistent associations of this group with recurrent pregnancy loss or stillbirth, and the American College of Obstetricians and Gynecologists (ACOG) argues that the prophylactic dose treatment of LMWH to improve live birth rates in women with hereditary thrombophilia and a history of pregnancy loss shows no benefit when compared to no treatment.^4^

 Similarly, the ACOG does not recommend screening for hereditary thrombophilia in women who have obstetric complications, including REM, placental abruption, IUGR, or PE, because it is unclear whether anticoagulation reduces the recurrence of these events.^4^ In this study, the rates of PE and preterm births in patients with DVT and negative aPL results were 5.3% and 11.3%, which were similar to those of the Brazilian general population,^7^ and the mean birth weight was 3.225 g (± 565). The results demonstrated that these patients had a favorable gestational prognosis that did not differ significantly from that of low-risk pregnancies. 

 One of the limitations of this study is the difficulty in reproducing the study in other locations, since it was carried out at a single center. The retrospective nature of the study could also lead to data loss in medical records. In addition, the absence of a control group of pregnant women without any diseases and the possibility of testing antibodies during pregnancy may lead to false-negative results. 

 However, this study was conducted in a specialized high-risk prenatal center with a large number of patients with venous thrombosis with and without APS, forming a homogeneous population despite its rarity. To reduce confounding factors, patients with systemic lupus erythematosus were excluded, allowing for a clearer analysis of the impact of primary APS on pregnancy. 

## CONCLUSIONS

 The presence of aPLs in patients with a history of DVT is associated with worse pregnancy outcomes, whereas patients with venous thrombosis and negative aPL results have gestational results similar to those in the general population. Our study suggests that investigation for APS is important for adequate follow-up of pregnant women with a history of DVT. Furthermore, the treatment of patients with APS during pregnancy can significantly improve live birth rates; however, other obstetric complications, such as PE, IUGR, and low birthweight, remain high despite treatment. 
